# Reconfigurable, Temperature Resilient Phase‐Change Metasurfaces Fabricated via High Throughput Nanoimprinting Lithography

**DOI:** 10.1002/advs.202521515

**Published:** 2026-03-26

**Authors:** Carlota de Ruiz de Galarreta, Yinghao Zhao, Jose Mendoza‐Carreño, José M. Caicedo, José Santiso, C. David Wright, M. Isabel Alonso, Jiafang Li, Agustin Mihi

**Affiliations:** ^1^ Institute of Materials Science of Barcelona ICMAB‐CSIC Bellaterra Spain; ^2^ Centre for Metamaterials Research & Innovation University of Exeter Exeter UK; ^3^ State Key Laboratory of Chips and Systems for Advanced Light Field Display School of Physics, Beijing Institute of Technology Beijing China; ^4^ Catalan Institute of Nanoscience and Nanotechnology (ICN2) CSIC and The Barcelona Institute of Science and Technology, Campus UAB Bellaterra Spain

**Keywords:** chalcogenide phase‐change materials, chirality, nanoimprint lithography, porous materials, reconfigurable metasurfaces, thin films

## Abstract

The combination of metasurfaces with chalcogenide phase‐change materials is a highly promising route toward the development of multifunctional and reconfigurable nanophotonic devices. However, their transition into real‐world devices is hindered by several technological challenges. This includes, amongst others, the lack of large area photonic architectures produced via scalable nanofabrication methods as required for free‐space photonic applications, along with the ability to withstand the high temperatures needed for the phase‐change process. In this work, we present a scalable nanofabrication strategy for the production of reconfigurable metasurfaces based on high‐throughput, large‐area nanoimprint lithography that is fully compatible with chalcogenide phase‐change materials processing. Our approach involves the direct imprinting of high‐melting‐point, thermally stable TiO_2_ nanoparticle pastes, followed by the deposition of an Sb_2_Se_3_ thin film as the phase‐change material active layer. The patterned TiO_2_ film enables the creation of thermally robust metasurfaces, overcoming the limitations of conventional polymer‐based nanoimprinting techniques. The versatility of our approach is showcased by producing phase‐change devices with two distinct functionalities: (*i*) metasurfaces with tunable spectral band switching and amplitude modulation capabilities across the near‐ to mid‐infrared, and (*ii*) reconfigurable chiral metasurfaces, whose chiroptical activity can be switched between the visible and the near‐infrared. Experimental results show excellent agreement with numerical simulations and reveal high uniformity across large areas. This work provides a universal, thermally robust and scalable platform for the production of reconfigurable metasurfaces based on phase‐change materials, paving the way to low‐cost, photonic devices with reconfigurable optical responses that could be extended far beyond the applications demonstrated here.

## Abstract 2

Large‐area, reconfigurable phase‐change metasurfaces are fabricated using a high‐throughput nanoimprint lithography approach based on thermally robust mesoporous TiO_2_. Integration with low‐loss Sb_2_Se_3_ enables reversible spectral tuning, high‐contrast infrared modulation, and dual‐band chiral switching from the visible to the near‐infrared. This scalable, polymer‐free platform overcomes key thermal and fabrication limitations, paving the way toward low‐cost, large‐area reconfigurable nanophotonic devices.

## 1 Introduction

As the field of nanophotonics keeps expanding and maturing, research efforts have turned to the development of active and reconfigurable devices, whose optical behavior can be altered or modified post‐fabrication via the incorporation of materials capable of reversibly responding to external physical stimuli [1, 2]. Among the various strategies available to yield reconfigurable nanophotonic devices, the use of chalcogenide phase‐change materials (PCMs) has emerged as one of the strongest approaches due to their inherent low power consumption, fast switching speeds, and abrupt refractive index contrast between their fully reversible amorphous and crystalline phases [3, 4, 5, 6]. Traditionally employed in commercial non‐volatile optical and electrical memories [4], chalcogenide PCMs such as the archetypal alloy Ge_2_Sb_2_Te_5_ (GST) can reversibly transition between amorphous, crystalline, and intermediate states through thermal stimuli. Such stimuli are typically provided by time and power‐controlled electrical or optical pulses, supplied, in the case of nanophotonic devices, by resistive heaters, or laser excitation, respectively [3, 4, 5, 7, 8]. In particular, crystallisation is achieved by heating the material above its crystallisation temperature, while amorphisation involves a melting step, followed up by a rapid cooling of the molten phase (up to tens of °C per nanosecond for GST) to prevent recrystallisation [3, 4]. As a result of their remarkable physical properties, PCMs have over the past decade PCMs been successfully combined with nanophotonic architectures such as metasurfaces, integrated photonic circuits, or thin film multilayer stacks [2, 3, 5]. This has led to the development of devices with diverse functionalities including non‐mechanical beam steering [8, 9], reconfigurable metalensing [10, 11, 12], switchable image pre‐processors [13, 14], amplitude modulation [15, 16], tunable structural colouring and filtering [17, 18], or neuromorphic photonic computing to name but a few [19, 20].

**FIGURE 1:**
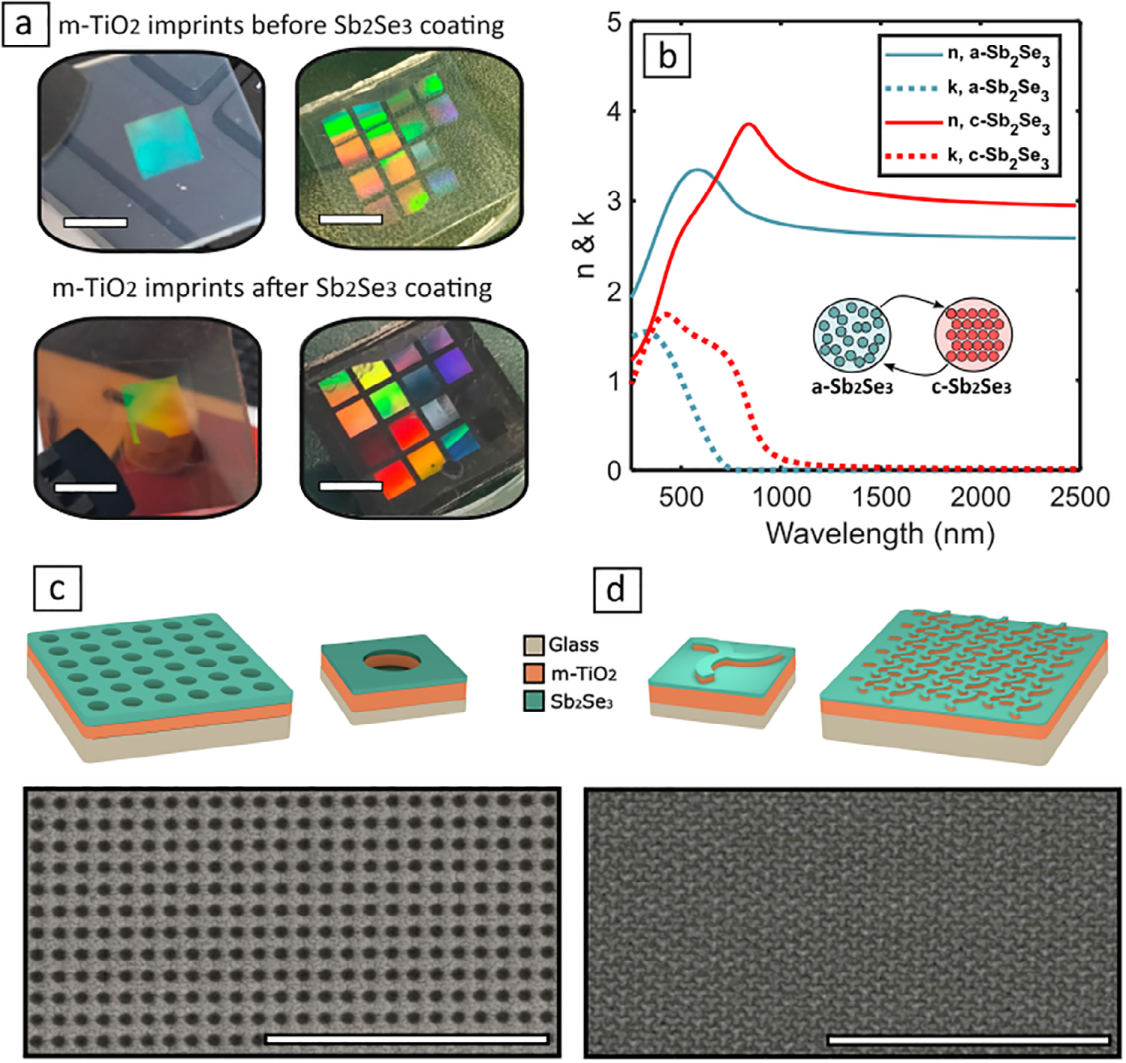
Schematics, optical properties, and morphology of large‐scale nanoimprinted phase‐change metasurfaces (a) Optical images of m‐TiO_2_ imprinted arrays before (top) and after (bottom) deposition of Sb_2_Se_3_ (Scale bars are 1 cm). (b) Refractive index (n, solid lines) and extinction coefficient (k, dashed lines) of our amorphous (a‐Sb_2_Se_3_, blue) and crystalline (c‐Sb_2_Se_3_, red) films, obtained by spectroscopic ellipsometry. (c) Schematic illustration (top) and SEM image (bottom) of ordered hole m‐TiO_2_ arrays coated with Sb_2_Se_3_. (d) Schematic illustration (top) and SEM image (bottom) of hexagonal m‐TiO_2_ triskelia arrays coated with Sb_2_Se_3_. Scale bars in SEM images is 10 µm.

Although significant advances in the field have been made in recent years, the successful integration of PCMs into reconfigurable metasurfaces for real‐world devices remains a formidable challenge due to several engineering crossroads arising from such a technological combination [2, 3], including thermal and chemical incompatibilities, or device endurance, to name a few [21]. Another critical ‐and often overlooked‐ aspect of such technology is the combination of PCMs with large‐scale, cost‐efficient, and large area nanofabrication technologies [2]. To date, PCM‐based metasurfaces have been mostly fabricated using electron beam lithography combined with reactive ion etching and/or lift‐off techniques [3, 7, 8, 9, 12, 13, 16]: processes that offer excellent versatility, pattern fidelity and resolution, but at the same time suffer from low throughput, limited scalability, and high cost. Alternative microfabrication techniques based on direct laser writing of crystalline meta‐atom arrays on amorphous PCM films have been recently explored and experimentally validated in the mid‐ to far‐infrared [22, 23]. However, although highly scalable, this approach is limited in terms of design versatility and optical performance; while it also suffers from reduced resolution arising from the diffraction limit and optical penetration depth of PCMs, making it not so well suited to the production of metasurfaces operating in the near‐infrared and visible spectral range. In this context, nanoimprint lithography (NIL) offers a compelling alternative due to its simplified implementation, low cost, high resolution, and suitability for large‐area patterning [24, 25]. Nevertheless, and in spite of its excellent scalability potential, to date the use of NIL toward large‐scale production of reconfigurable phase‐change metasurfaces has remained unexplored. In this work, we address this key omission by introducing a high‐throughput, low‐cost NIL nanofabrication route suitable for patterning PCM‐based reconfigurable metasurfaces over large areas. Our approach enables the direct nanoimprinting of mesoporous TiO_2_ (m‐TiO_2_) paste made of anatase nanoparticles, for subsequent deposition of Sb_2_Se_3_ thin films (Figure 1a). The Sb_2_Se_3_ coating provides a high refractive index tunable environment through its amorphous and crystalline phase transitions (Figure 1b). On the other hand, the nanoimprinted m‐TiO_2_ backbone not only offers excellent resolution and pattern homogeneity over large areas, but crucially –and contrary to conventional polymer‐based resins— it grants high thermal stability capable of withstanding temperatures of at least 1000°C for 1H [26]. This aspect is critical from a device functionality point of view, as temperatures required for a successful cycling of chalcogenide PCMs can go above 170°C and 600°C for crystallisation and re‐amorphisation processes, respectively [3, 4]. To illustrate the potential and versatility of our approach, we have developed a set of Sb_2_Se_3_‐based reconfigurable metasurfaces, showcasing two distinct functionalities. As depicted in Figure 1c, the first type of metasurfaces consists of square arrays of microholes supporting high‐quality‐factor resonances, whose resonant spectral position can be switched by changing the phase of the Sb_2_Se_3_ layer between amorphous, crystalline, and intermediate states. This design provides reconfigurable filtering and modulation across the near and mid‐ infrared, with experimental absolute modulation depths as high as 60% in both reflection and transmission. The second type of metasurface studied is constituted of a hexagonal array of triskelion motifs engraved in the m‐TiO_2_ and coated with Sb_2_Se_3_ (Figure 1d)_._ This chiral array exhibits a strong resonant chiroptical response that can be switched between the visible and the near‐infrared when the Sb_2_Se_3_ layer transitions from amorphous to crystalline. All the devices developed and optically characterised showed good agreement with numerical simulations, confirming the effectiveness and versatility of our approach. Therefore, we believe our approach could be generalized toward the development of scalable and low‐cost phase‐change metasurfaces with high temperature resilience, for additional functionalities to those shown here, including, but not limited to, active beam steering, dynamic colour displays, reconfigurable optical analogue computing and more

## 2 Results and Discussion

### 2.1 Fabrication and Architecture of Nanoimprinted PCM Metasurfaces

**FIGURE 2:**
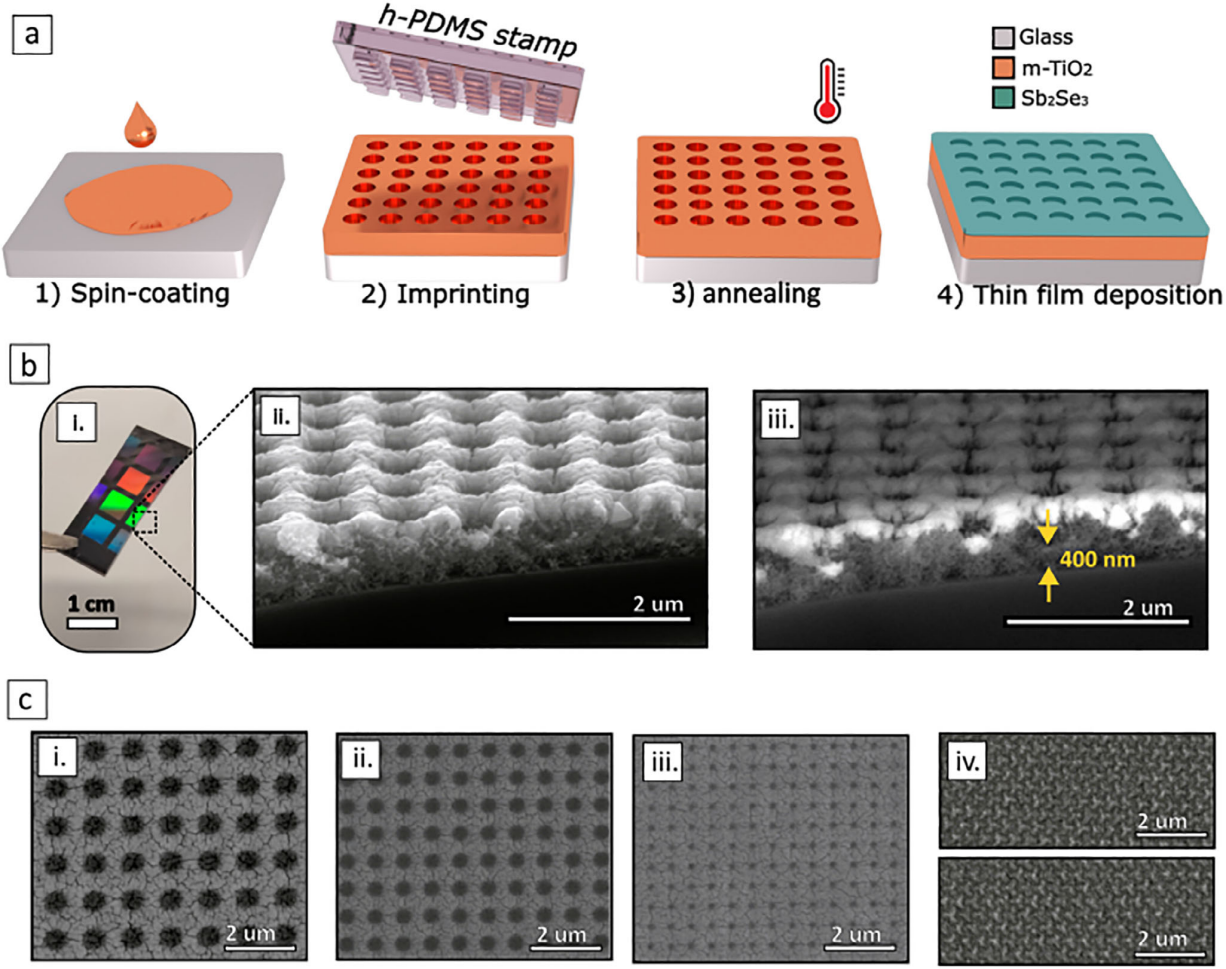
Fabrication and structural characterisation of nanoimprinted PCM metasurfaces (a) Flowchart of the fabrication process summarizing the key steps. (b) Cross sectional device characterisation: (i) Optical image of a patterned chip showing multiple device areas prepared for SEM imaging (ii) Cross‐sectional SEM image of a completed device, and (iii) its corresponding back scattering electron detection version, revealing fine details about the porous m‐TiO_2_ layer, sandwiched between the Sb_2_Se_3_ coating (brighter region) and the glass substrate (darker region). (c) Top‐view SEM images showing a variety of devices fabricated and optically characterised in this work. (i–iii) Square arrays of holes with different lattice parameters and hole diameters. (iv.) Hexagonal arrays of left‐handed (top) and right‐handed (bottom) triskelion motifs.

Figure 2a summarizes the fabrication process undertaken for the metasurfaces showcased in this work. First, a commercial TiO_2_ paste (used herein as the NIL resist) made of 20 nm diameter anatase nanoparticles was vigorously diluted in ethanol in a 1:3 (w/w) ratio, and then spin coated onto clean glass substrates at 2000 rpm for 10 s, with an acceleration of 1000 rpm.s^−1^. Next, pre‐patterned hybrid stamps made of hard‐PDMS (h‐PDMS) and soft‐PDMS (s‐PDMS) as the backbone were used to imprint the desired patterns onto the TiO_2_/ethanol spin‐coated films on a hot plate at 125°C for 10 min. The patterned films were then baked at 500°C for one hour to eliminate the organic fraction of the paste, leading to the m‐TiO_2_ arrays. Note we choose an m‐TiO_2_ annealing temperature of T = 500°C as this is the highest temperature available in our hot plate, but annealing routines at values of up to 1000°C have also been successfully employed [26], making the herein fabrication process particularly attractive for systems and/or applications which require to withstand high temperatures. Moreover, around 800°C, anatase nanoparticles transition to rutile [26, 27], providing a higher refractive index medium which potentially offers additional design degrees of freedom to our approach. Following the fabrication flow shown in Figure 2a, after obtaining the m‐TiO_2_ nanostructured backbone, an Sb_2_Se_3_ layer was deposited on top via pulsed laser deposition. We selected Sb_2_Se_3_ as the phase‐change material due to its low optical losses in both amorphous and crystalline states across the near‐ to mid‐ IR when compared to more traditional GeSbTe ternary alloys [28, 29, 30]. A detailed account of the materials, protocols and instruments used can be found at the *Experimental section* (Sections 4.1–4.4).

The cross‐sectional SEM inspection (Figure 2b) of one of the fabricated devices shows the presence of both the PCM coating and the m‐TiO2 backbone. In particular, the back‐scattering electron image shown in Figure 2b (iii) reveals an m‐TiO2 residual layer of ∼400 nm, which was found to be present in all the devices. Moreover, to verify the stoichiometry of the Sb_2_Se_3_ overlayer, we performed energy‐dispersive spectroscopy (EDS). Results from such measurements can be found in the Section S1, where we show atomic percentages of Sb (42.64%) and Se (57.36%). This corresponds to an atomic ratio of *Sb*: *Se* ≈ 2: 3 within the EDS error, indicating that the stoichiometry of the Sb2Se3 target was properly transferred to the film.

Finally, in Figure 2c we present a set of PCM metasurface architectures fabricated and optically characterised in the next sections, elucidating the versatility and geometrical scalability of our fabrication strategy. In particular, in Figure 2c (i–iii), we show several square arrays of microholes with a variety of lattice parameters and hole diameters, which were employed for spectral band switches and modulators across the near to mid‐infrared (in Section 2.1). On the other hand, Figure 2c (iv) shows left‐handed and right‐handed hexagonal arrays of triskelion motifs, whose chiroptical activity can be switched between the visible and infrared regimes, as further discussed in Section 2.3.

### 2.2 Phase‐Change Scalable Metasurfaces for Infrared Band Switching and Modulation

The first set of PCM metasurfaces studied is intended for infrared reconfigurable band switching and modulation. This functionality has been successfully exploited in different ways and spectral bands for key cutting‐edge applications such as active color displays [31, 32], multispectral thermal imaging [33], or reconfigurable image processing [13, 34]. However, to date, their fabrication has always relied on expensive, cleanroom‐based, conventional electron‐beam lithography carried out over small areas, thereby limiting their scalability potential.

**FIGURE 3:**
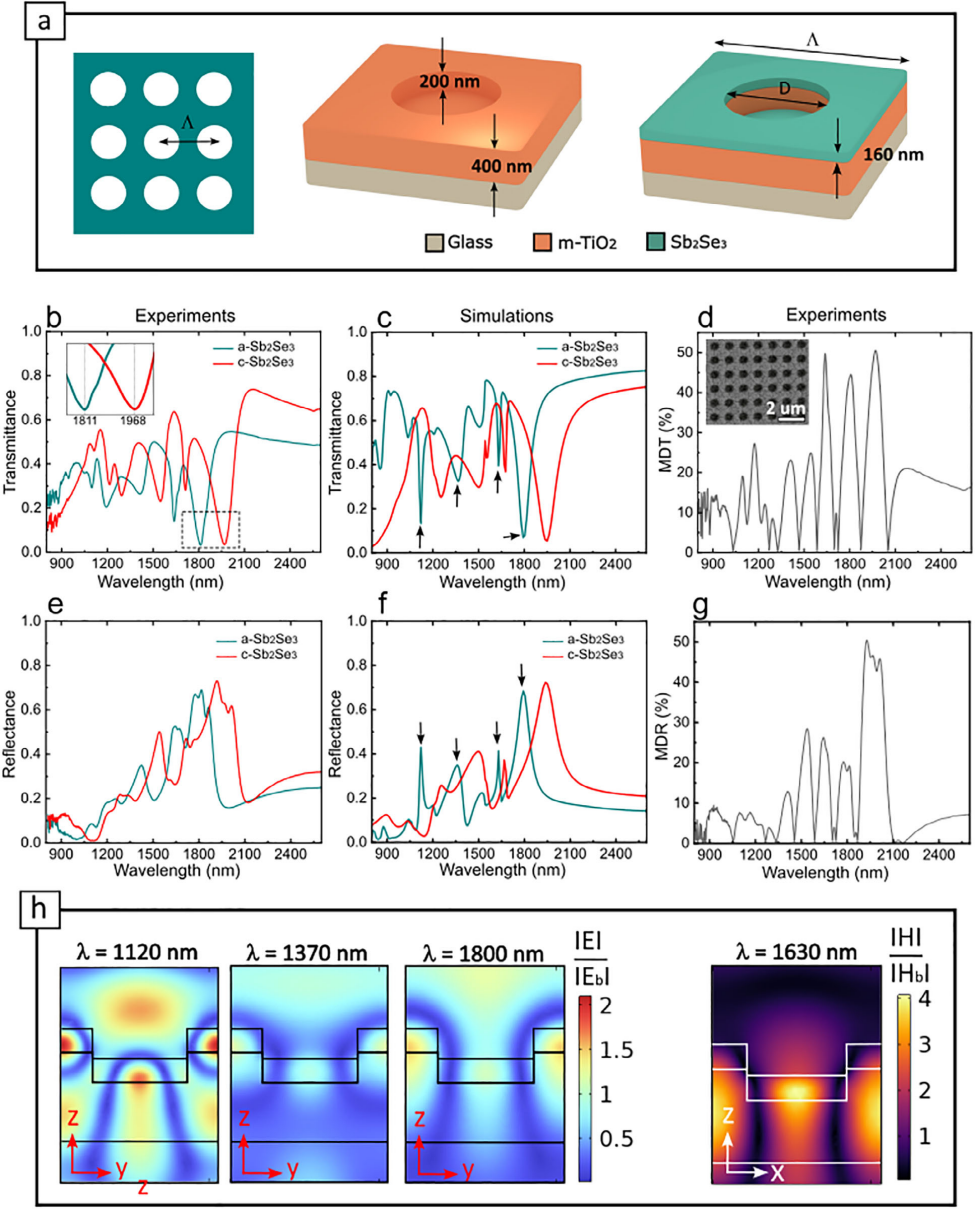
Nanoimprinted Sb_2_Se_3_‐based metasurfaces for spectral filtering and modulation. (a) Schematic and geometrical characteristics of our metasurface unit cell, consisting of a mesoporous TiO_2_ (m‐TiO_2_) square array of holes, coated with an Sb_2_Se_3_ layer. (b,c) Experimental (b) and simulated (c) transmittance spectra for amorphous (a‐Sb_2_Se_3_, turquoise) and crystalline (c‐Sb_2_Se_3_, red) states for a device with D = 0.65 µm, and Λ = 1.06 µm (d) Experimentally obtained modulation depth in transmission (MDT), revealing absolute modulation values up to 50% (inset shows an SEM image of the device). (e,f) Experimental (e) and calculated (f) reflectance spectra of the same device for a‐Sb_2_Se_3_ and c‐Sb_2_Se_3_ states. (g) Experimentally obtained modulation depth in reflection (MDR) (h) Simulated enhanced field distributions (|E|/|E_b_| and |H|/|H_b_| for TE and TM modes, respectively) at representative resonant wavelengths identified with black arrows in (c) and (f), for excitation with x‐polarised light.

A generic unit cell of the proposed device is depicted in Figure 3a. It consists of an m‐TiO_2_ slab lying on a glass substrate, and imprinted with holes of 200 nm depth, forming a square array with an m‐TiO_2_ residual layer of thickness 400 nm. An Sb_2_Se_3_ film of thickness *t_Sb2Se3_* = 160 nm covers the holes, providing a high refractive index tunable dielectric environment to the device upon transitioning the Sb_2_Se_3_ from its amorphous to crystalline states. The Sb_2_Se_3_ film is protected with a 15 nm thick Al_2_O_3_ layer (not represented for simplicity) to prevent its oxidation [8, 21]. These geometrical parameters were kept constant for all the devices, while a set of arrays with different hole diameters (D) and lattice constants (Λ) was fabricated.

Figure 3b displays the measured transmittance spectra of a device with *D* = 0.64 µm, and *Λ* = 1.06 µm for both amorphous (a‐Sb_2_Se_3,_ turquoise curve) and crystalline (c‐Sb_2_Se_3,_ red curve) states. Crystallisation of the device was carried out using a hot plate (as described in Section 4.6). As can be observed, the metasurface exhibits a set of sharp resonances manifested as transmittance dips across the near‐ to mid‐ IR spectral range (see Section 4.7 for details on the optical measurements). These resonances are spectrally shifted by Δλ ∼ 175 nm upon crystallisation of the phase‐change layer (see inset in Figure 3b), due to an abrupt increase of its refractive index, accompanied by a minor decrease of the quality factor as a result of the non‐negligible absorption coefficient of crystalline Sb_2_Se_3_ (as displayed in Figure 1b). Our experimental results are also in good agreement with finite element simulations carried in COMSOL multiphysics, and shown in Figure 3c for comparison (details, material properties and boundary conditions employed in our finite element models can be found in Section 4.5). Finally, in Figure 3d we show the absolute modulation depth of the device in transmission (MDT), obtained from the experimental values as [15]

(1) $$MDT\left(\lambda \right)=\left|T_{am}\left(\lambda \right)-T_{cr}\left(\lambda \right)\right|\;$$

where *T_am_* and *T_cr_* are the transmittance spectra for the metasurface with amorphous and crystalline Sb_2_Se_3,_ respectively. Absolute experimental modulation values as high as 50% can be observed at different sharp spectral bands across the mid IR. Figure 3e,f display the measured and simulated reflectance spectra of the same device for both a‐Sb_2_Se_3_ and c‐Sb_2_Se_3_. As in previous results from transmission measurements, a set of resonances can be identified, here, however, manifested as complementary sharp reflectance peaks, indicating that the transmission dips observed in Figure 3b–d correspond to light being specularly reflected to free‐space by the metasurface. Modulation depths achieved in reflection (MDR) were found to be in line with transmission values (i.e. around 50%, as revealed in Figure 3e). To elucidate the effect of the periodic patterning in our films, optical measurements were also carried out outside the patterned region (i.e. at regions with unpatterned multilayer stacks made of glass, 400 nm of m‐TiO_2,_ 160 nm of Sb_2_Se_3_ and 15 nm of Al_2_O_3_). As shown in the Section S2, these measurements revealed the presence of a single vertical Fabry‐Pérot resonance coming from interferences at the Sb_2_Se_3_ and m‐TiO_2_ layers. Therefore, the rich, multiresonant optical behaviour of our metasurfaces arises exclusively from the as‐designed geometrical architecture. In particular, the Sb_2_Se_3_ film endows the structure with a high refractive index, generating Mie‐like resonators, while the periodic perturbation coming from the TiO_2_ underlayer adds extra momentum to launch hybrid leaky‐guided modes (LGM) inside the m‐TiO_2_ residual layer, the latter behaving as an intermediate refractive index waveguide (n_m‐TiO2_ ∼1.6) forming an asymmetric system cladded by the lower refractive index glass substrate (n_glass_∼1.5). Further insights into the nature of these resonant modes were obtained via analysis of the electromagnetic field enhancement at the four resonant wavelengths highlighted with black arrows in Figure 3c–f. The enhanced electromagnetic field distribution under x‐polarised light of such modes is depicted in Figure 3h. These were calculated as |E|/ |E_b_|and |H |/ |H_b_|, where |E_b_| and |H_b_|are the maxima of the modulus of the electric and magnetic fields, taken from air domains far away from the device). Following a modified Fabry–Pérot model [35, 36], we classify the transverse electric and magnetic modes as TE(*m, n, l*) and TM(*m, n, l*), respectively, where *m*, *n* and *l* represent the mode quantum numbers or number of electric/magnetic antinodes along the *x*, *y*, and *z* axes. Based on this definition, we find three LGM resonances, which can be classified as TE(1,3,2) at *λ* = 1120 nm, TE(1,3,1) at *λ* = 1800 nm, and TM(3,1,1) at *λ* = 1630 nm. An additional Mie‐like electric dipole resonance can be observed at *λ* = 1370 nm, where electric fields are weakly confined inside the high refractive Sb_2_Se_3_ domains only. This less intense electric field confinement is also manifested as a lower quality factor in the reflectance and transmittance spectra (shown in Figure 3c,f) when compared to the other higher Q‐factor LGM resonances, whose electric and magnetic field confinements are also visibly higher (see Figure 3h). We note that, in contrast to ref [37]–where electromagnetic confinement occurs within air domains— the field localisation here occurs predominantly inside finite‐sized Sb_2_Se_3_ dielectric domains, thus our localised modes are identified as standard Mie‐like resonances proper from high index particles (rather than Mie‐void resonances as in [37]).

**FIGURE 4:**
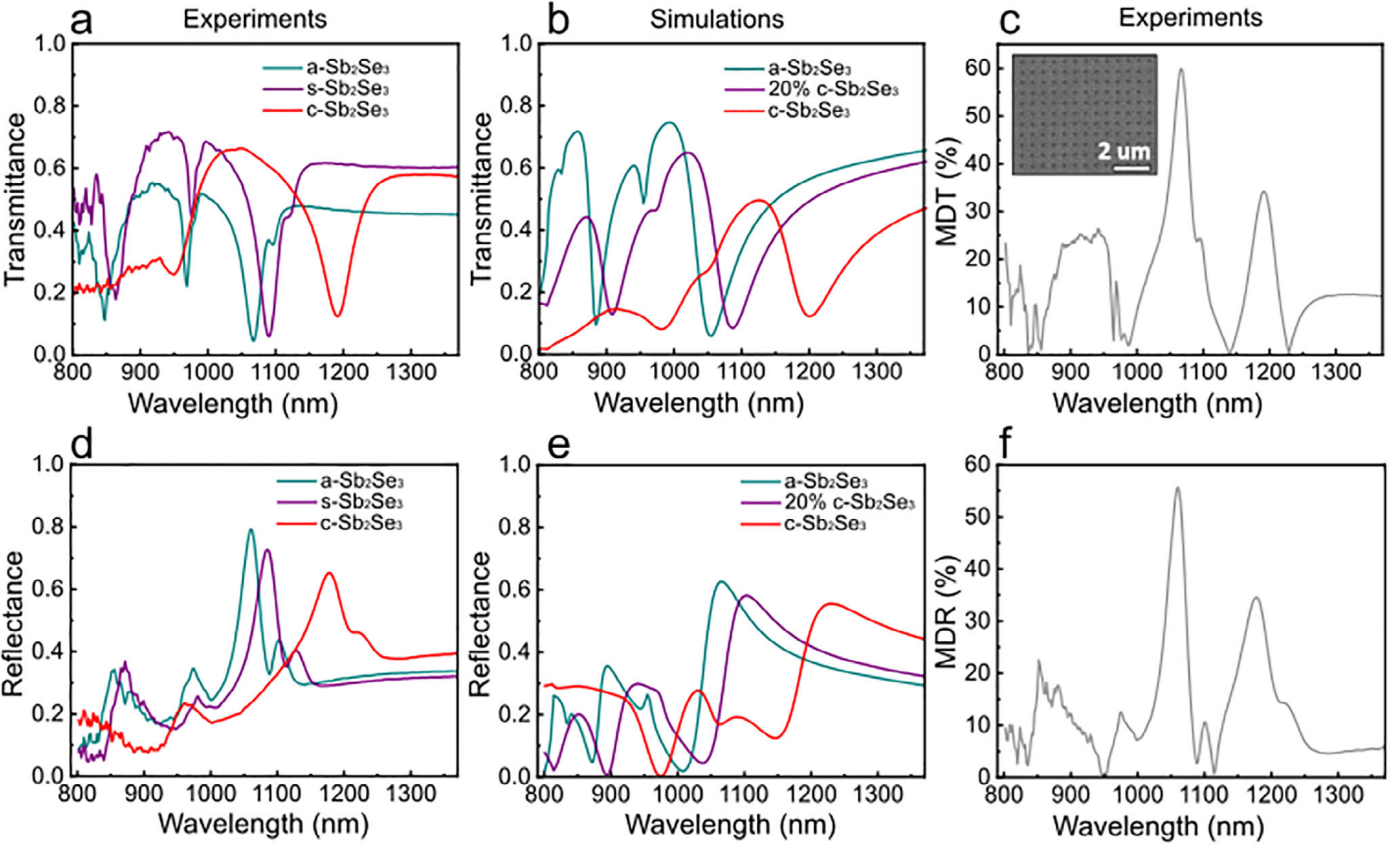
(a,b) Experimental (a) and simulated (b) transmittance spectra for amorphous (a‐Sb_2_Se_3_, turquoise), semicrystalline (s‐Sb_2_Se_3_, purple) and crystalline (c‐Sb_2_Se_3_, red) states for a device with D = 0.26 µm, and Λ = 0.56 µm. (c) Experimentally obtained modulation depth in transmission (MDT), revealing absolute modulation values up to 50% (inset shows an SEM image of the device). (d,e) Experimental (d) and calculated (e) reflectance spectra of the same device for a‐Sb_2_Se_3_, s‐ Sb_2_Se_3_, and c‐Sb_2_Se_3_ states. (f) Experimentally obtained modulation depth in reflection (MDR).

To explore the spatial resolution achievable with our PCM metasurface fabrication routine, we fabricated and tested devices with significantly smaller geometrical features, such as those shown in Figure 2c (iii) (with a period Λ = 0.56 µm, and a hole diameter D = 0.26 µm). Moreover, multilevel switching capabilities were also investigated via partial crystallisation experiments (described in Section 4.6). In Figure 4a, we show the measured transmittance spectra for amorphous (turquoise), semicrystalline (purple) and crystalline (red) Sb_2_Se_3_. Due to a significant reduction of the metasurface dimensions with respect to the previous device, the multiresonant behaviour is shifted toward the blue, falling now in the near‐infrared regime. Our fabrication routine can therefore be employed to customise the position of the LGM resonances across the IR, by simply changing the geometrical parameters of the h‐PDMS/s‐PDMS hybrid stamps. As it can be observed, a progressive crystallisation of the Sb_2_Se_3_ layer results in a gradual red‐shift of the modes –arising from a gradual increase of its refractive index— and paving the way to a multilevel tunable filtering functionality, rather than a simpler binary configuration. This behaviour is well‐reproduced in simulations (shown in 4b), where intermediate crystalline states were calculated using the COMSOL Multiphysics effective medium approach tool (based on the Maxwell‐Garnett approximation). By fitting the resonant peak positions of the experimental semicrystalline spectrum, we determined that the partially crystallised experimental state corresponds to approximately 20% of crystallisation fraction in our model. Importantly, the device exhibits absolute modulation depths in transmission (MDT) of up to 60% (Figure 4c): values comparable to those obtained in previous larger‐geometry devices. In Figure 4d,e we present the corresponding experimental and calculated reflectance spectra for the same device, which here again were found to be almost complementary to transmittance spectra (i.e. with transmission resonant dips corresponding to light being mostly specularly reflected by the metasurface). Finally, to assess the robustness of our fabrication approach, we performed reproducibility tests on identical devices produced on different days. As discussed in Section S4, reflection and transmission spectra were highly consistent from device to device, suggesting **reproducibility and reliability** of our approach. Additional PCM metasurfaces with varied geometrical parameters and intermediate crystallisation states were also successfully fabricated and tested (Section S3), further demonstrating the versatility of our method for realising PCM metasurfaces with customised reconfigurable infrared band‐switching and modulation capabilities over large areas.

### 2.3 Dual‐Band Chiral Phase‐Change Metasurfaces

**FIGURE 5:**
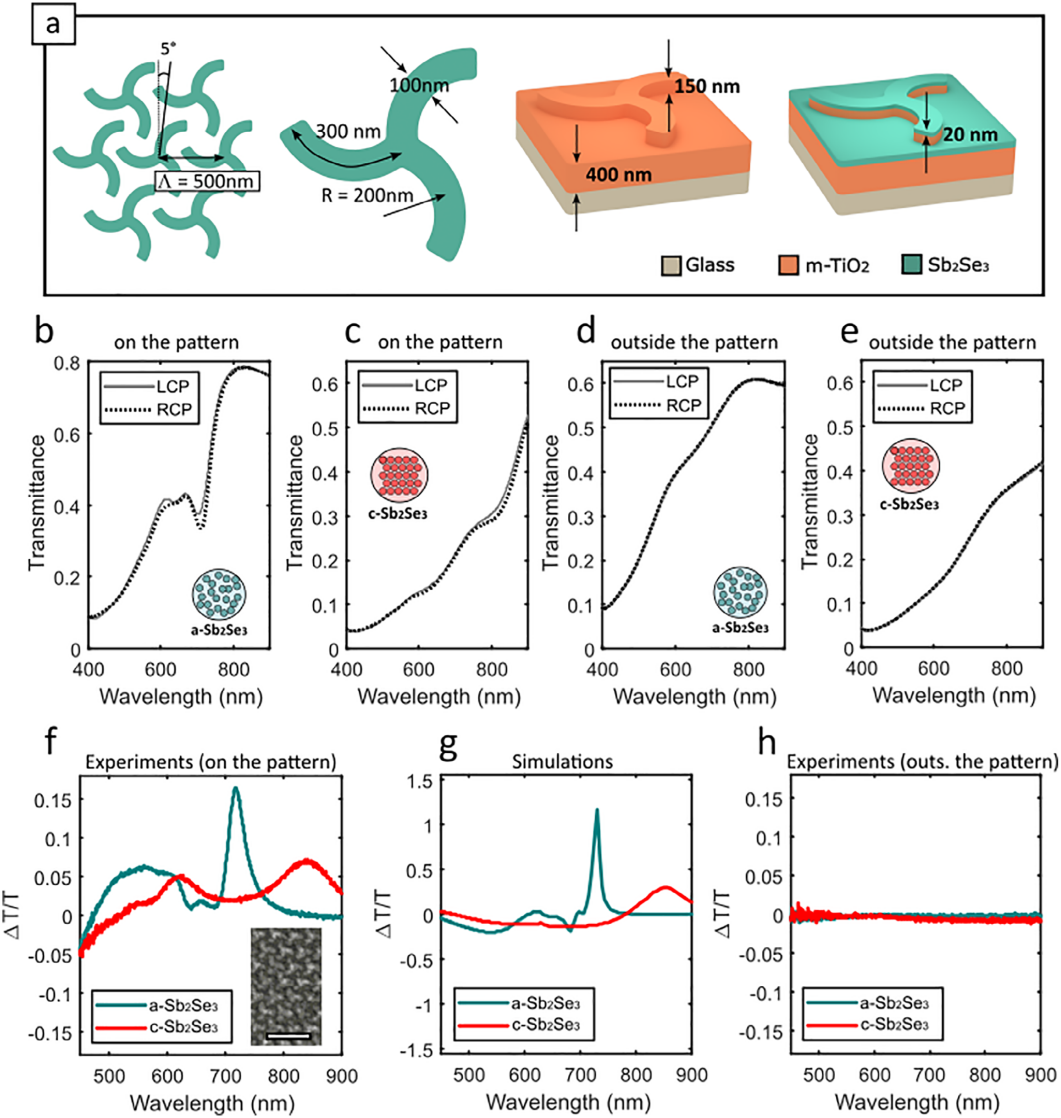
Reconfigurable chiral metasurfaces based on nanoimprinted m‐TiO_2_ triskelion motifs coated with Sb_2_Se_3_. (a) Schematics of our phase‐change chiral metasurface architectures and their geometrical parameters (b,c). Experimental transmittance spectra under left (LCP, grey solid) and right (RCP, black dashed) circularly polarised light for amorphous (b) and crystalline (c) Sb_2_Se_3_ states, taken from left‐handed triskelion metasurfaces. (d,e) Control transmittance spectra measured outside the patterned area, revealing identical results for both polarisations and Sb_2_Se_3_ phases. (f) Experimentally obtained dissymmetry factor (ΔT/T) for amorphous (turquoise) and crystalline (red) states. The inset shows an SEM image of the device (scalebar 1 µm). (g) Simulated dissymmetry factor for amorphous and crystalline Sb_2_Se_3_ (h) Experimental dissymmetry factor outside the metasurface, confirming the absence of chiroptical response.

To further explore the potential and versatility of our fabrication approach, we fabricated metasurfaces with reconfigurable chiroptical activity based on left‐ and right‐handed arrays of nanoimprinted triskelion motifs. We choose triskelion‐based arrays due to their lack of mirror symmetry, which prevents them from being mapped onto their mirror image by any combination of rotations or translations. Triskelia‐based metasurfaces have been widely exploited as nanophotonic architectures for (static) giant circular dichroism, chiral second harmonic generation, and chiral light emission [38, 39, 40, 41]. However, to date, the combination of chiral metasurfaces with PCMs toward reconfigurable chiroptical activity has remained largely underexplored, with only a few demonstrations reported in the mid‐infrared and relying on complicated 3D nanofabrication protocols [42, 43]. In contrast, we here demonstrate a simplified architecture compatible with our scalable fabrication strategy, which allows the tailoring of the chiroptical response of our phase‐change metasurfaces down to the red part of the visible spectrum, enabling the spectral tuning of its chiroptical activity from the red into the near‐infrared upon crystallisation. This capability unlocks new opportunities, e.g. polarisation‐sensitive reconfigurable imaging or optical communications. Figure 5a shows the schematics and geometrical parameters of our phase‐change chiral nanophotonic architecture. The triskelia motifs have an arm width of 100 nm, a length of 300 nm, and an arm curvature radius of R = 200 nm. The unit cell is arranged in a hexagonal configuration, with the triskelia patterns rotated by 5°, and a lattice constant of Λ = 500 nm. Rotation of the triskelia motifs with respect to the lattice was found to improve the circular dichroism, as rigorously discussed in ref [38]. In analogy to the hole arrays discussed in the previous section, an m‐TiO_2_ residual layer of 400 nm with a triskelion height of 160 nm was chosen. The entire structure is then capped with 20 nm of Sb_2_Se_3_ and protected with 15 nm of Al_2_O_3_. Based on numerical simulations (Section S5), an Sb_2_Se_3_ thickness of 20 nm was deemed a good value to achieve a pronounced spectral shift of the chiroptical response between the visible and infrared upon switching the PCM between amorphous and crystalline states. These design choices enable the metasurfaces to operate as a visible‐to‐infrared chiral switch.

In order to quantify the chiroptical activity of our metasurfaces, we rely on the dissymetry factor *ΔT/T* defined as [44, 45]

(2) $$\frac{\Delta T}{T}=2\frac{T_{LCP}-T_{RCP}}{T_{LCP}+T_{RCP}}$$

where *T_LCP_* and *T_RCP_* are the ballistic transmittance spectra for left and right circularly polarised light, respectively. In Figure 5b,c, we show the measured transmittance spectra of our left‐handed chiral phase‐change metasurfaces for LCP (grey solid line) and RCP light (dashed black line), and for amorphous (Figure 5b) and crystalline (Figure 5c) states. These measurements were carried out at normal incidence in a customised optical setup, whose details are described in detail in Section 4.7. Clear differences between LCP and RCP can be already observed in the transmittance spectra when compared to measurements taken outside the patterns (i.e. where transmittance was found to be essentially identical for both polarisations and both states, as revealed by Figure 5d,e). In Figure 5f, we show the experimental dissymetry factor, where a resonant dissymetry factor peak of ΔT/T∼0.15 can be observed around λ = 715 nm when the Sb_2_Se_3_ layer is in its amorphous phase (i.e. almost at the red edge of the visible spectrum). After crystallisation, this peak undergoes a remarkable red shift of Δλ∼ 120 nm, relocating it in the near infrared (specifically around λ = 840 nm). Besides, and in line with previous results from hole‐arrays, the crystalline state exhibits a broader peak as a result of resonance damping introduced by the non‐negligible absorption coefficient in crystalline Sb_2_Se_3._ Crucially, despite this spectral broadening, the chiral switching functionality remains robust, spanning from the visible to the near‐infrared upon crystallisation. This behaviour is further reproduced by our numerical simulations performed in Lumerical (see Section 4.4. for simulation details), and displayed in Figure 5g, revealing once again a good qualitative agreement with experimental results. We note that while qualitative agreement of the dissimetry factor between simulations and experiments is excellent, our experiments show a dissimetry factor which is significantly smaller than in simulations. We attribute the quantitative discrepancy between the simulated and experimental dissymmetry factors to limitations in the resolution of the fabricated triskelion arms. Highmagnification SEM imaging (Figure S6) reveals that some triskelia arms are distorted. Given that the arm width is only 100 nm (see Figure 5a), we can conclude that we are here at the limit of resolution of our patterning technique (i.e. x5 times the particle size of our TiO2 paste, which is 20 nm). As a comparison, the experimental dissymetry factor outside the samples was also computed (Figure 5h), where no chiroptical activity was observed for any of the Sb_2_Se_3_ structural phases, further confirming that the dissymetry resonant peaks are coming exclusively from the phase‐change nanophotonic architecture presented herein. Finally, analogous experiments were also carried out on right‐handed triskelion metasurfaces, with results shown in the supplementary information (Section S6). As expected and revealed by Figure S7, the measurements show a mirrored spectral behavior, that is, a dual‐band chiroptical response upon switching from amorphous to crystalline states, but with opposite signs of the dissymmetry factor. This is fully consistent with the enantiomeric nature of the structures, and further demonstrates the visible‐to‐infrared chiral switching capabilities of our scalable phase‐change metasurfaces.

## 3 Conclusion

In summary, we have demonstrated a novel, scalable, and thermally robust routine for the fabrication of reconfigurable phase‐change metasurfaces by strategically combining high‐throughput direct nanoimprint lithography of m‐TiO_2_ architectures with Sb_2_Se_3_ coatings. Due to the high thermal resilience of m‐TiO_2_, our approach overcomes the limitations of low‐melting‐point polymer‐based NIL, paving the way to the integration of phase‐change materials into large‐area devices capable of withstanding the characteristic high temperatures required for the phase‐change transitions. In addition, as shown in Table S1 (Section S8), our fabrication method provides a unique combination of low cost, large‐area coverage, and high‐resolution patterning within a single process compared to other state‐of‐the‐art techniques such as direct laser processing, electron beam lithography or focusing ion beam milling. The versatility and resolution of the proposed technique have been illustrated through a set of devices having two different functionalities: infrared band switches and modulators with absolute modulation depths as high as 60%, and chiral metasurfaces with dissymetry factors of up to ΔT/T ∼0.15, whose chiral properties can be switched between the visible and the near‐infrared. To the best of our knowledge, this work also provides the first experimental demonstration of chiral phase‐change metasurfaces operating in the visible and near‐infrared regimes [42, 43]. Overall, the excellent agreement between experiments and simulations, combined with geometrical scalability and reproducibility across multiple devices, highlights the robustness of our platform.

Finally, although reversible switching of the Sb_2_Se_3_ active layer (from amorphous to crystalline) was not performed in this study, recent works have demonstrated laserinduced cycling of Sb_2_Se_3_ and Sb_2_S_3_ films—both continuous [29]. and nanostructured [9]—on thermally insulating   [10]. and thermally conductive substrates [29].

In addition, it is worth noting that the “all‐dielectric” nature of the metasurface architectures reported here may a priori seem to limit the practical use of resistive heaters for in situ switching of the phase‐change layer, as recently reported in refs [7, 46]. However, in Figure S8 from the supplementary information, we show how imprinting of m‐TiO_2_ can also be readily performed on conductive materials such as metals, thereby opening the door to future cheap and cost‐efficient PCM‐based metasurfaces with in situ electrical switching capabilities employing metal resistive heaters. Therefore, our approach can be generalised and further explored toward the creation of cheap, and thermally robust phase‐change reconfigurable metasurfaces over larger areas, and with exciting functionalities additional to those shown herein, including reconfigurable beam steering devices, image pre‐processors, or tunable color displays, to name but a few.

## 4 Experimental Section

### 4.1 Materials

Acetone, Isopropanol, Ethanol, and glass substrates were purchased from Labbox. The soft PDMS kit (Sylgard 184) was purchased from Dow Corning (Midland, Michigan, USA). The compounds for synthesizing hard PDMS were acquired from Gelest (USA). Perfluorooctyl‐trichlorosilane for the master's silanization was acquired from Merck. Titania paste (90‐T) based on 20 nm diameter anatase nanoparticles was purchased from Dyesol (greatcellsolar), Elanora. The Sb_2_Se_3_ target for thin film depositions was purchased from Testbourne (Netherlands).

### 4.2 Fabrication of h‐PDMS/s‐PDMS Stamps

Working stamps for imprinting of m‐TiO_2_ were based on hybrid h‐PDMS/s‐PDMS composites. To prepare PDMS stamps with pillars for the fabrication of hole arrays, the main silicon masters (consisting of arrays of holes) were cleaned in acetone and isopropyl alcohol (10 min ultrasonication each), and subsequently splashed with deionised water followed by drying with compressed air. Next, and in order to prevent permanent adhesion of PDMS to silicon, clean masters were silanized with 12 µl of perfluorooctyl‐trichlorosilane, under low vacuum in a desiccator for 30 min. The silanized masters were then post‐baked for 20 min at 150°C on a hot plate. After the masters were clean and silanized, a s‐PDMS 10:1 mixture (monomer: curing agent) was mixed vigorously using a hand stirrer, and left degassing for one and a half hours. Meanwhile, the h‐PDMs were prepared by mixing 0.85 g vinylmethylsiloxane, 25 µL of 1,3,5,7‐tetracetylcyclosilane, 2 µL of Pt catalyst, 275 µL of hydroxyl siloxane, and 1 mL of toluene under vigorous mixing assisted by a magnetic stirrer. The h‐PDMS was then drop cast onto the master, and spread using a compressed air gun. The drop casting and air spreading process was repeated for a couple of times. The substrates were left for 30 min at room temperature, followed by an annealing procedure on a hot plate at 60°C for 1 H. Finally, degassed s‐PDMS was poured onto the masters and thermally cured at 100°C for approximately 1H. For preparing h‐PDMS/s‐PDMS with holes to make triskelion arrays of pillars, the process was essentially the same, except for the fact that the main working masters were made of Ormostamp pillar replicas obtained from main silicon masters consisting of arrays of triskelion holes.

### 4.3 Thin Film Deposition

Sb_2_Se_3_ thin films were prepared by pulsed laser deposition from pure (99.99%) Sb_2_Se_3_ one‐inch targets. A KrF excimer laser (λ  =  248 nm, COMPex 201, Lambda Physik, Coherent, Göttingen) was used for the deposition at RT, with a pulse duration of 20 ns, a repetition rate of 10 Hz, an energy of 50 mJ and a spot size of 2.6 mm^2^, resulting in an energy fluence of about 1.9 J/cm^2^ The Argon pressure inside the vacuum chamber was set to 0.13 mbar, and the distance from target to substrate of about 70 mm. These conditions resulted in a growth rate of 0.37 Å/pulse.

### 4.4 Spectroscopic Ellipsometry

Ellipsometry measurements were taken with a SOPRALAB GES5E rotating polariser ellipsometer at several angles of incidence in a spectral range from 1.2 to 5.5 eV (ca. 250–1030 nm).  The analysis of ellipsometric data was done using an in‐house code. The fitting model considered the test sample consisting of a SiO_2_/Si substrate with the Sb_2_Se_3_ film deposited on top and Al_2_O_3_ capping. To represent the n and k of the films, we tested different analytic line shapes. The as‐deposited amorphous films could be well described by a sum of two Tauc‐Lorentz oscillators, indicating a fundamental band gap of 1.60 eV (775 nm). However, this model was not valid for the crystallised sample, fitted using a sum of a constant and two modified harmonic oscillators, with the lowest energy one centred at 1.46 eV (849 nm). The refractive index extrapolated to the infrared range with these models agreed with the FTIR measured reflectance.

### 4.5 Numerical Calculations

Finite element calculations for the hole arrays were performed employing the commercial software package COMSOL Multiphysics (RF module), via rigourous solving of Maxwell's equations. To mimic infinite arrays of elements, Floquet periodic boundary conditions were applied to the lateral domains of the unit cell shown in Figure 3a, whereas top and bottom domains were truncated using periodic ports. The refractive index of glass substrates and m‐TiO_2_ were assumed to be constant across the entire IR due to their low dispersion in this spectral regime, with n_glass_ = 1.5 and n_m‐TiO2_ = 1.6) [47]. The complex refractive indices of amorphous and crystalline Sb_2_Se_3_ shown in Figure 1b were obtained by ellipsometry measurements. Triskelia arrays were simulated employing the commercial software (FDTD Lumerical from ANSYS). The m‐TiO_2_ triskelia array had an arm width and height of 100 and 160 nm, respectively. A conformal coating layer of 20 nm was added of the phase change materials with the same optical properties used for the hole arrays. A pair of plane waves was injected with a phase delay of ±90° for LCP and RCP. The ballistic transmittance of both circular polarisations was collected using a power monitor to mimic the experimental characterisation for low NA objectives.

### 4.6 Phase‐Change Annealing Experiments

Partial crystallisation of Sb_2_Se_3_‐based metasurfaces was achieved by annealing the devices on a hot plate for 1 min at the material crystallisation temperature (170°C) [17]. Full crystallisation was carried out by increasing the annealing time from 1 min to 5 min. Additional crystallisation tests were carried out at higher temperatures (i.e. 190°C), but no significant differences on the device optical properties were found. However, and in line with previous studies, annealing at temperatures above 190°C and/or longer times was found to severely degrade the metasurface performance. This behavior has been observed previously and has been attributed to selenium loss and diffusion through the capping layers (here, Al_2_O_3_), which can result in a loss of the optimum stoichiometry [30, 48].

### 4.7 Reflectance and Transmittance Optical Measurements

Reflectance and transmittance spectra were acquired through FTIR measurements (BRUKER Vertex 70 equipped with a microscope and an HgCdTe cooled detector). A low numerical aperture objective (NA 0.1) was used to minimise the light cones of excitation and collection, thereby preventing possible resonance broadening and/or generation of additional modes due to angular dispersion arising from periodic structures.

For reflection measurements, a reference baseline was taken by collecting the signal from a silver mirror with an average reflectance of R = 97%, which was then used to normalise the raw spectra from the samples. Air was used as a reference baseline for transmission measurements. Both types of measurements were carried out with unpolarised light.

### 4.8 Circular Dichroism Measurements in Transmission

Circular dichroism of triskelia‐based phase‐change metasurfaces was measured employing a home‐built optical setup [45]. Briefly, the excitation part was based on a fibre‐coupled white lamp (Ocean Optics, HL‐2000‐HP, FL, USA) with an output reflective collimator made of protected silver (RC08SMA‐P01, Thorlabs). A Glan‐Thompson linear polariser (GTH10M, Thorlabs) was placed before the sample in order to obtain linearly polarised light, and an achromatic quarter‐wave plate (SAQWP05M‐700) was subsequently employed to obtain either right or left circularly polarised light (i.e. by mounting the quarter‐wave plate at an angle of ±90° with respect to the axis of the input linearly polarised light). The sample was then placed between two low NA objectives (NA 0.1), here again to reduce the cone of excitation and collection angles and ensure quasi‐normal incidence. Finally, light was collected employing a second reflective collimator (protected silver) fiber coupled to a spectrometer (Ocean Optics, QEPro‐FL). Transmittance spectra for RCP and LCP were taken by normalizing the raw signal from the sample to the raw signal collected from air for each polarisation state. The dark signal from the spectrometer was subtracted from every raw measurement before normalisation.

### 4.9 SEM/EDS Measurements

SEM and EDS measurements were taken with a Quanta 200 ESEM FEG from FEI. Images were acquired under low vacuum (60 Pa) and with an acceleration voltage of 15 kV.

## Conflicts of Interest

The authors declare no conflicts of interest.

## Supporting information




**Supporting File**: advs74607‐sup‐0001‐SuppMat.docx.

## Data Availability

The data that support the findings of this study are available from the corresponding author upon reasonable request.
