# Supplementary material for: Reconfigurable, Temperature Resilient Phase‐Change Metasurfaces Fabricated via High Throughput Nanoimprinting Lithography
Source: Adv Sci (Weinh). 2026 Mar 26;13(27):e21515. doi: 10.1002/advs.202521515 (PMC13170255; doi:10.1002/advs.202521515)
Supplement: Supplementary file 1 — Supporting File: advs74607‐sup‐0001‐SuppMat.docx. [file ADVS-13-e21515-s001.docx]

Supplementary information for

Reconfigurable, Temperature Resilient Phase-Change Metasurfaces Fabricated via High Throughput Nanoimprinting Lithography

Carlota Ruiz de Galarreta+*^1,3^, Yinghao Zhao+^1,2^, Jose Mendoza-Carreño^1^, José M Caicedo^4^, José Santiso^4^, C David Wright^3^, M Isabel Alonso^1^, Jiafang Li^2^, Agustin Mihi*^1^

*1 Institute of Materials Science of Barcelona ICMAB-CSIC; Campus UAB, Bellaterra*, Spain
^2^ State Key Laboratory of Chips and Systems for Advanced Light Field Display, School of Physics, Beijing Institute of Technology, Beijing 100081, China
^3^ Centre for Metamaterials Research & Innovation, University of Exeter, Exeter EX44QF, UK
^4^ Catalan Institute of Nanoscience and Nanotechnology (ICN2), CSIC and The Barcelona Institute of Science and Technology (BIST), Campus UAB, Bellaterra, Spain.

E-mail: *[cruiz@icmab.es](mailto:cruiz@icmab.es), *amihi@icmab.es

+ Y.Z and C.RdG contributed equally

**Supporting Information**

S1. SEM/EDS analysis of the Sb_2_Se_3_ films

S2. Transmission and reflection measurements of Glass/m-TiO2/Sb2Se3 unpatterned multilayer stacks

S3. Optical characterization of additional phase-change hole arrays for band switching and modulation.

S4. Device reproducibility tests

S5. Dependence of the dissymmetry factor with the Sb2Se3 layer thickness:

S6. SEM characterisation of triskelia and optical characterization of the right-triskelion enantiomer

S7. Fabrication of m-TiO2 nanostructures on different substrates: Aluminum
S8. Comparison of our fabrication routine to other techniques:

**S1: SEM/EDS analysis**

To confirm the elemental composition of our devices and stoichiometry of the Sb_2_Se_3_ thin films, Energy Dispersive X-ray Spectroscopy (EDS) analysis was performed in conjunction with Scanning Electron Microscopy (SEM). As shown in Figure S1(top), specific x-rays corresponding to elements such as O, Si, Na and Mg can be attributed to the glass substrate, Ti and O to the m-TiO_2_ layer, and Se and Sb to the Sb_2_Se_3_ upper film. To assess the stoichiometry of the Sb_2_Se_3_ film, we proceed to eliminate all the elements from the analysis except Sb and Se. As revealed by Figure S1(bottom) atomic percentages of Sb (42.64%) and Se (57.36%) were measured. This corresponds to an atomic ratio close to $Sb:Se\approx2:3$ within the EDS error.


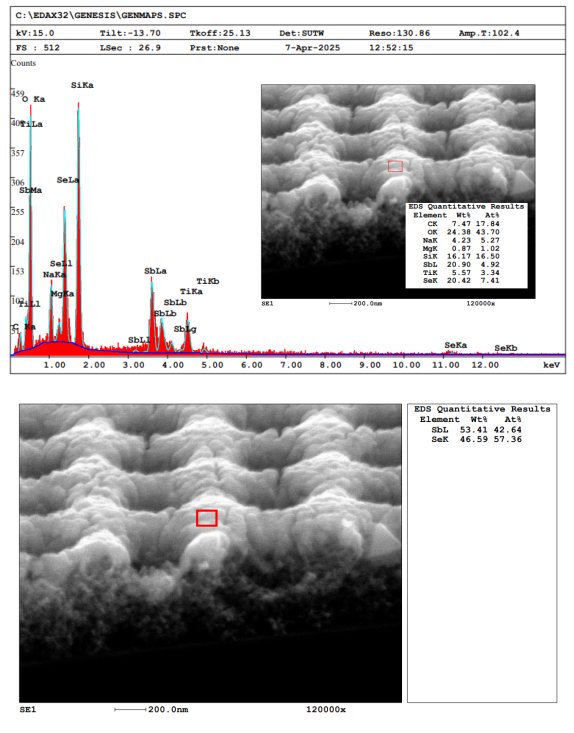


Figure S 1 TOP: EDS spectrum showing all the elements present in our devices. BOTTOM: analysis of the atomic percentage of Sb and Se, excluding the rest of elements.

**S2. Transmission and reflection measurements of Glass/m-TiO_2_/Sb_2_Se_3_ unpatterned multilayer stacks**

The influence of the proposed nanophotonic architecture on the transmittance and reflectance properties was investigated via optical measurements carried out outside the hole-arrays (i.e. where only an unpatterned multilayer stack made of glass, m-TiO_2_ (400nm) and Sb_2_Se_3_ (160 nm) is present). In Figure S2, we show this results for crystalline Sb_2_Se_3_ compared to simulations, revealing here again an excellent agreement. As it can be seen –and contrary to the spectra measured on patterned devices exhibiting multiple resonances (see Figure 3 from the main paper)—, a single Fabry-Pérot resonance can be observed when the periodic perturbation of the underlying m-TiO_2_ layer is not present.


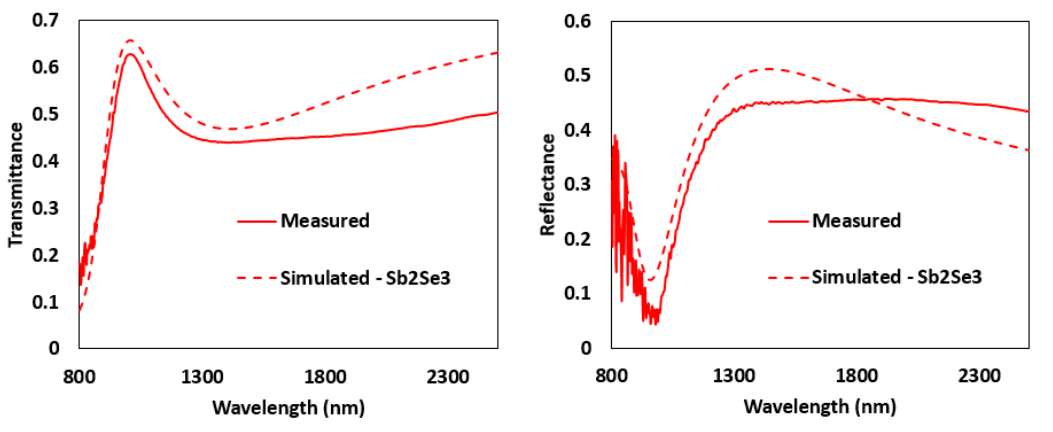


Figure S 2. Measured transmittance (left) and reflectance (right) spectra (solid lines) carried out otuside the patterned area, and compared to simulations (dashed lines).

**S3. Optical characterization of additional phase-change hole arrays for band switching and modulation.**

Figure S3 shows the transmittance and reflectance spectra for fully amorphous, fully crystalline, and partially crystalline Sb_2_Se_3_ of a device with a lattice parameter Λ = 0.75 μm, and a hole diameter D = 0.48 μm (corresponding to the SEM image shown in Figure 2c(ii) from the main text). The experimental modulation depth in both transmission and reflection is also plotted for reference. Here again, a good agreement between simulations and experiments can be observed.


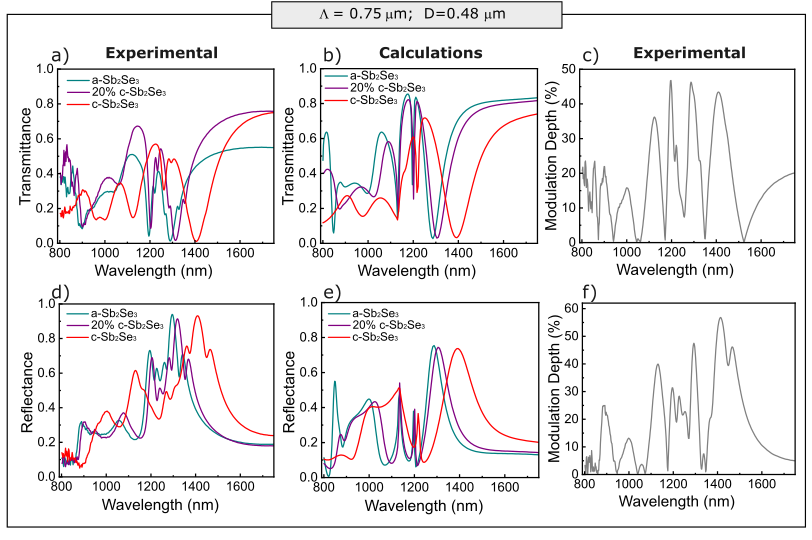


Figure S 3 Experimental and calculated optical responses for a device with Λ = 0.75 μm and D = 0.48 μm, (a,b) Transmittance spectra: (a) experimental and (b) calculated for amorphous Sb_2_Se_3_​, 20% crystallised Sb_2_Se_3_​, and crystalline Sb_2_Se_3_​, (c) Experimental modulation depth in transmittance- (d,e) Reflectance spectra: (d) experimental and (e) calculated. (f) Experimental Modulation depth calculated in reflectance.

**S4. Fabrication reproducibility tests**

To evaluate the reproducibility of our fabrication approach, we measured the reflectance and transmittance spectra of two identical devices. Each device was fabricated in separate batches, with TiO₂ solutions prepared on different days and Sb₂Se₃ deposited on different days as well.

Figure S4 shows reproducibility tests performed on two distinct hole-array device geometries. In Figure S4 (left), results are presented for a device with a period Λ = 0.56 μm, a hole diameter D = 0.28 μm, and an Sb₂Se₃ thickness of 160 nm in the amorphous phase. In Figure S4 (right), results are shown for devices with Λ = 1.66 μm, D = 1.00 μm, and an Sb₂Se₃ thickness of 160 nm.

Overall, a good agreement between the devices was observed, with only minor variations in the positions of the resonant peaks. We attribute these small deviations to thickness gradients in the Sb₂Se₃ films, which can be caused by pulsed laser deposition due to the Gaussian profile of the laser beam (thereby Gaussian spatial dependence of the target ablation), as well as the by the angular distribution of the plasma plume.^[1]^ Such variations could be minimized by employing alternative thin-film deposition methods that provide better uniformity over larger areas, such as thermal evaporation or magnetron sputtering.^[2,3]^

| 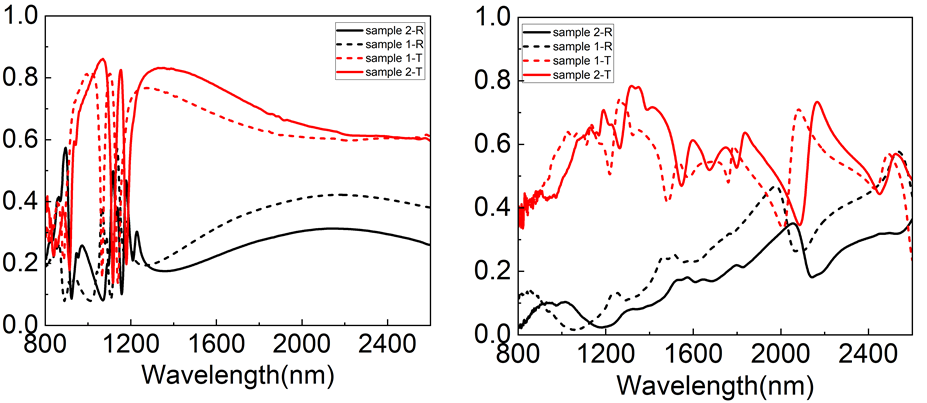  Figure S 4 Fabrication reproducibility tests for two hole-array device geometries. (Left) Device with period Λ = 0.58 μm, hole diameter D = 0.3 μm, and Sb₂Se₃ thickness of 160 nm (amorphous phase). (Right) Device with period Λ = 1.66 μm, hole diameter D = 1.00 μm, and Sb₂Se₃ thickness of 160 nm. Reflectance (R) and transmittance (T) spectra are shown for two independently fabricated samples (1 and 2). |
| --- |

**S5. Dependence of the dissymmetry factor with the Sb2Se3 layer thickness:**

In Figure S5, we show the dissymmetry factor (ΔT/T, colorbar) of left-handed triskelion arrays as a function of the Sb_2_Se_3_ overlayer thickness, for amorphous (left) and crystalline (right) states. As it can be observed, increasing the thickness results in a gradual red-shift of the dissymmetry factor resonant peak, with thickness values above 40 nm exhibiting maxima in the infrared for both amorphous and crystalline states. In order to enable the visible to IR switch functionality, we therefore choose a thickness of 20 nm, where the dissymmetry factor peak falls around λ~700 nm (i.e. at the red edge of the visible spectrum) when the Sb_2_Se_3_ layer is amorphous, but switches to λ~850 nm (i.e. near IR) upon crystallization.


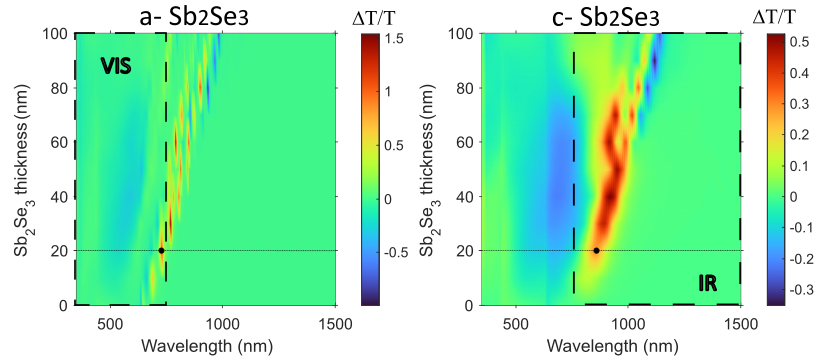


Figure S 5. Dissymetry factor ΔT/T spectra as a function of the Sb_2_Se_3_ layer thickness for amorphous (left) and crystalline (right) phases. Black dots show the dissymetry factor maxima for a thickness of 20nm, falling in the visible and near infrared for amorphous and crystalline states respectively.

**S6. SEM characterisation of triskelia and optical characterization of the right-triskelion enantiomer***A) Further SEM characterisation of triskelia*

From Figure S6, it can be seen that some of the triskelia arms are distorted after imprinting, indicating that the resolution limit of the proposed technique is around 100 nm (i.e. equal to the triskelia arm width).

**
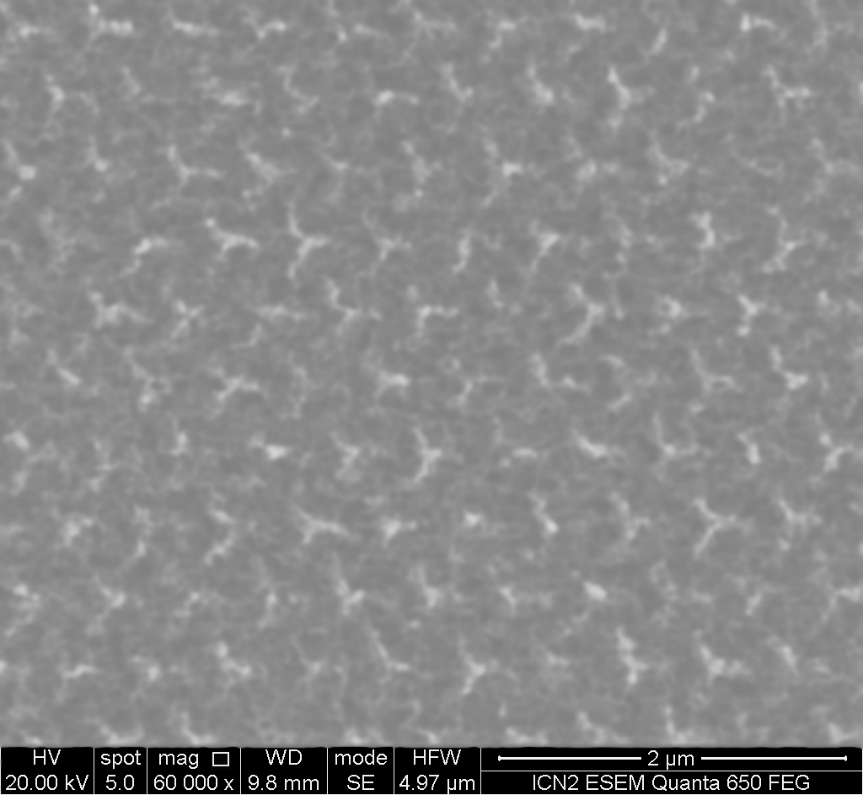
**

Figure S 6 Higher magnification SEM image of left-triskelia arrays

*B) Optical characterization of the right-triskelion enantiomer*

In addition to left-handed phase-change triskelion arrays, their enantiomer right-handed version (depicted in Figure S6a) a was also successfully fabricated and tested. In Figures S6b-c, we show the measured transmittance spectra or LCP and RCP light for both amorphous (Figure S6b) and crystalline (Figure S6c) phases. Using Eq. 2 from the main text, we then calculate the experimental dissymmetry factor of our arrays, displayed in Figure S6d. As it can be observed, in analogy to the phase-change left-handed arrays of triskelia, the right-handed version exhibits the same visible to infrared chiral switching capabilities, here however with opposite handedness (i.e. a negative dissymetry factor).


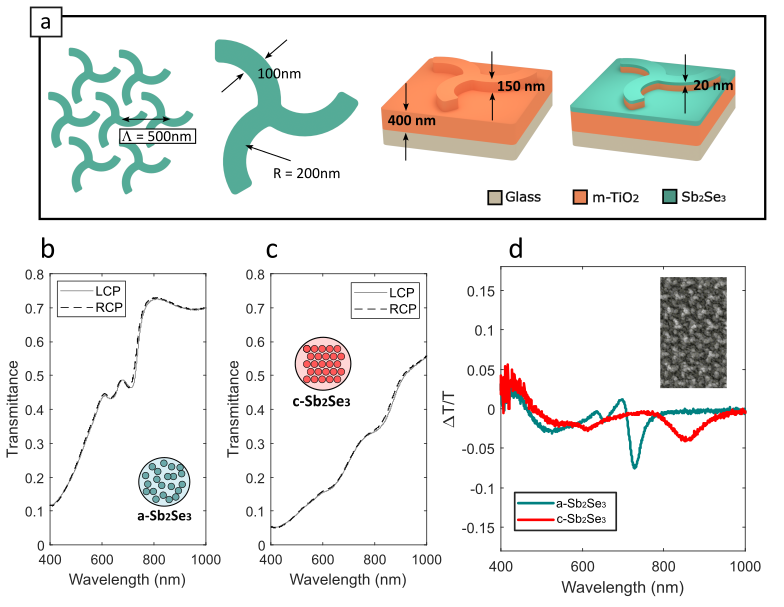


Figure S 7 (a) Schematic of the right-handed phase-change triskelion devices. (b–c) Experimental transmittance spectra under left (LCP, solid line) and right (RCP, dashed line) circularly polarised light for amorphous (b) and crystalline (c) states. (d) Experimental dissymmetry factor (ΔT/T) showing a clear spectral shift upon crystallization and a sign reversal compared to the left-handed enantiomer counterpart (shown in Figure 5 from the main text). Inset: SEM image of the fabricated right-handed array.

**S7. Fabrication of m-TiO2 nanostructures on alternative substrates: Aluminum**

To further investigate the versatility of our fabrication protocol –in particular the possibility of imprinting phase-change devices on top of conductive materials which could act as resistive in-situ heaters— we also performed m-TiO_2_ hole-array imprints on aluminum substrates, as schematically shown in Figure S7a. In Figures S7b-c, we show a couple of macroscopic views of different arrays, showing strong diffractive colors over of mm^2^ to cm^2^ (scalebars are 1 cm)

As confirmed by Figures S7d and S7e, high quality and highly homogeneous patterns can be readily obtained on alternative conductive substrates such as Aluminum.


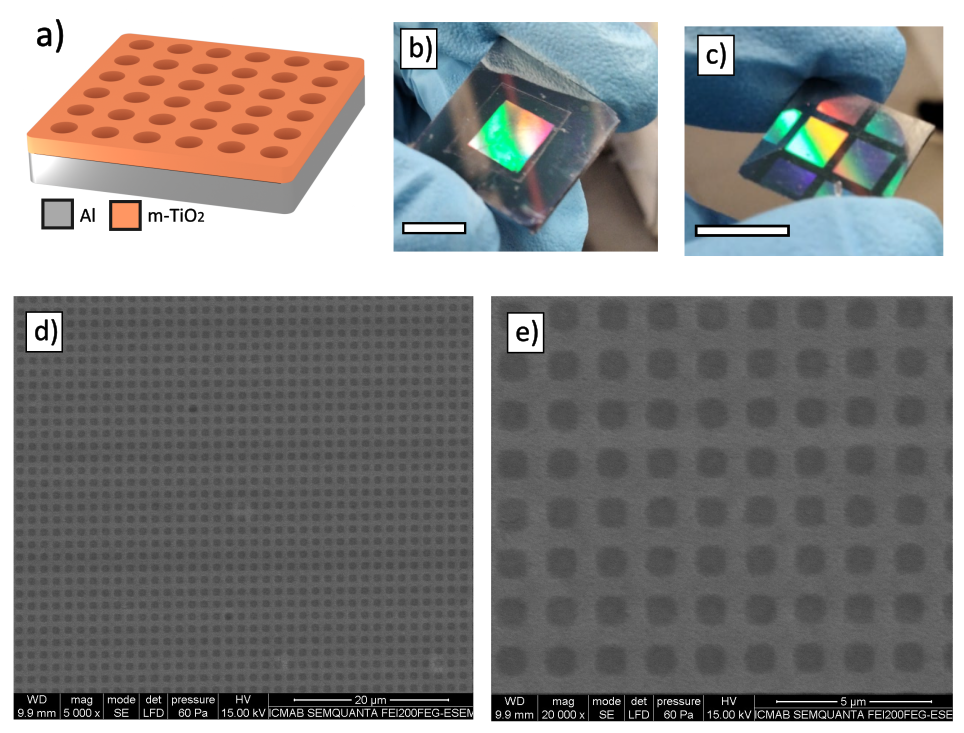


Figure S 8 (a) Schematic illustration of m-TiO₂ squared-hole-arrays directly imprinted on aluminum.
(b–c) Macroscopic optical images of a couple of fabricated samples showing strong diffractive colors over large areas ranging from mm² to cm² (scale bars = 1 cm). (d–e) SEM images at different magnifications confirming the high quality, uniformity, and homogeneity of m-TiO_2_ imprints on aluminum.

**S8. Comparison of our fabrication routine to other techniques:**

| Ref. | Patterning method | Large areas | Minimum feature size reported | Writing area and time | Cost |
| --- | --- | --- | --- | --- | --- |
| [4] | Direct laser ablation of thin gold layers | Yes | ~1 µm (at the limit) | 4 mm^2^, 20 min area dependent | low |
| [5] | Focusing Ion beam milling on silicon wafers | No | ~0.33 µm (potential for smaller sizes) | Not reported,  area dependent | high |
| [6] | electron beam lithography on PMMA masks + reactive ion etching of exposed Sb_2_Se_3_ areas | No | ~ 250 µm (potential for smaller sizes) | Not reported,  area dependent | high |
| This work | nanoimprint lithography on m-TiO_2_ paste | Yes | ~0.100 µm (limit of resolution) | 1 cm^2^, Instantaneous area independent | low |

Table S 1 Comparison of our fabrication routine to other techniques:

**References:**

[4] C. Ruiz de Galarreta, N. Casquero, E. Humphreys, J. Bertolotti, J. Solis, C. D. Wright, J. Siegel, *ACS Appl. Mater. Interfaces* 2022, 14, 2, 3446–3454.
[5] M. Hentschel, K. Koshelev, F. Sterl, S. Both, J. Karst, L. Shamsafar, T. Weiss, Y. Kivshar & H. Giessen*. Light Sci Appl* 12, 3 (2023)
[6] P. Moitra, Y. Wang, X. Liang, L. Lu, A. Poh, T. W.W. Mass, R. E. Simpson, A. I. Kuznetsov, R. Paniagua-Domínguez. *Adv. Mater.* 2023, 35, 2205367
